# Malignant pericardial effusion as a primary manifestation of metastatic colon cancer: a case report

**DOI:** 10.1186/s13256-021-03085-w

**Published:** 2021-10-28

**Authors:** Milena Brachmans Mascarenhas Neves, Mirella Velasco Stival, Yuri Costa Sarno Neves, Jordânia Gonçalves Pereira da Silva, Daniela Borges da Rocha Macedo, Bianca Mendes Carnevalli, Alana Moura Fé e Silva, Claudia Vaz de Melo Sette, Stephane Tomaz da Luz, Daniel de Iracema Gomes Cubero

**Affiliations:** 1 Faculdade de Medicina do ABC, Santo André, SP Brazil; 2 Instituto do Câncer do Estado de São Paulo, Hospital das Clinicas HCFMUSP, University of Sao Paulo, São Paulo, SP Brazil

**Keywords:** Case report, Pericardium effusion, Colon cancer, Metastasis

## Abstract

**Background:**

Pericardial neoplastic involvement is rarely related to primary tumors of the pericardium and is most often caused by spread from other primary sites, such as lung and breast carcinomas, hematological malignancies (lymphoma and leukemia), and melanoma. Although pericardial metastasis from infradiaphragmatic tumors (such as colon cancers) are rare and poorly described in literature, any neoplasm has the potential to metastasize to the pericardium and heart by either contiguity, lymphatic, or hematological spread.

**Case presentation:**

A 44-year-old previously healthy male Causasian patient had a sudden onset of dyspnea and wheezing. During investigation with echocardiogram, computed tomography and repeated pericardiocentesis, the cause of malignant pericardial effusion was confirmed as primary manifestation of metastatic colon cancer. The patient was treated with appropriate chemotherapy and presented satisfactory disease control.

**Conclusions:**

This report emphasizes the importance of considering the diagnostic hypothesis of occult neoplasia in a patient with pericardial effusion.

## Background

The prevalence of pericardial involvement in malignant neoplasms varies between 5% and 20% in autopsy series and clinical studies and is indicative of poor prognosis [1, 2]. Most common primary sites are lung and breast carcinomas, hematological malignancies, and melanoma [1, 3], although any tumor has the potential to metastasize to the heart by either contiguity or lymphatic or hematological dissemination. Metastasis from infradiaphragmatic tumors is rare and poorly described in the literature [4–7].

We report here the unique case of a patient who presented with symptomatic pericardial effusion as a rare initial clinical manifestation of metastatic colon adenocarcinoma. This case is important to highlight the need to consider neoplastic involvement of the pericardium sac even from distant malignancies and as the first manifestation of occult disease (onset of clinical symptoms) of a patient.

## Case presentation

**Fig. 1:**
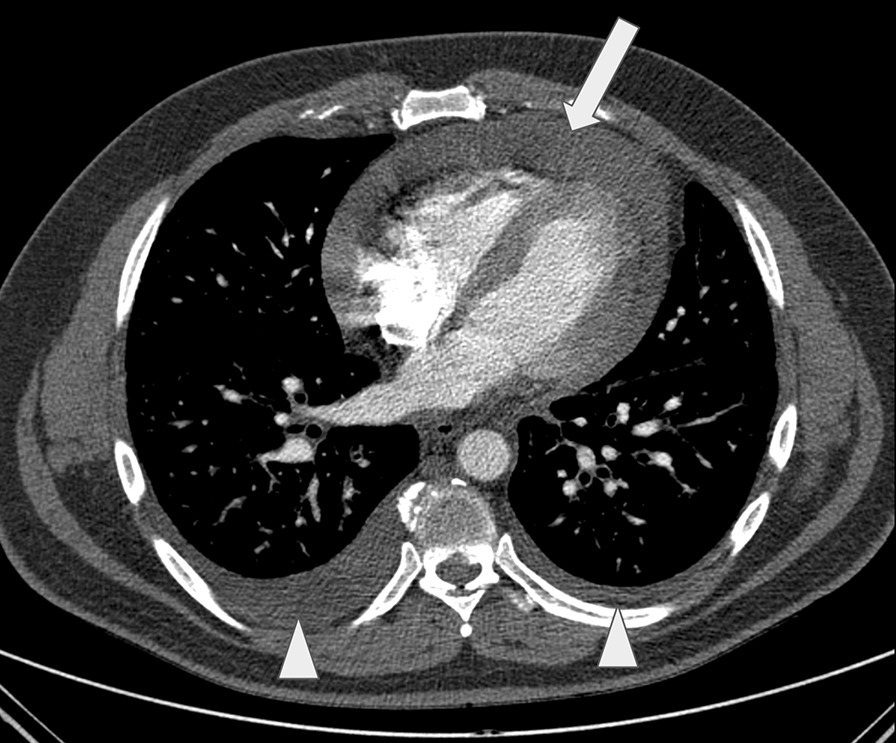
Contrast-enhanced chest computed tomography at the level of the inferior pulmonary vein shows moderate pericardial effusion (arrow) and small bilateral pleural fluid (arrowheads)

**Fig. 2:**
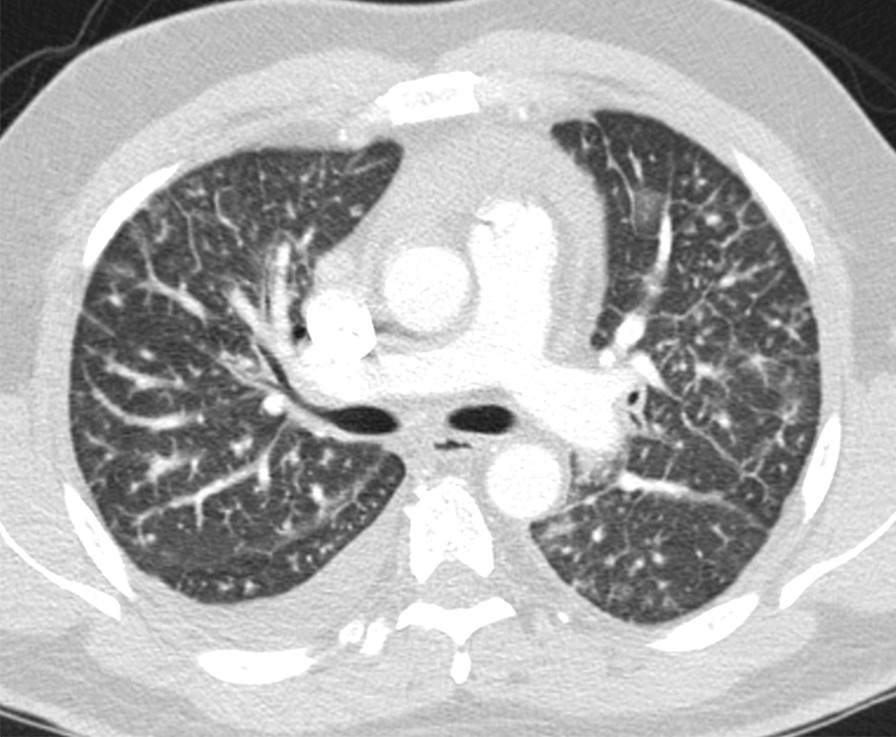
Chest computed tomography with lung window reveals diffuse bilateral septal thickening, compatible with venolymphatic congestion/pulmonary edema

**Fig. 3:**
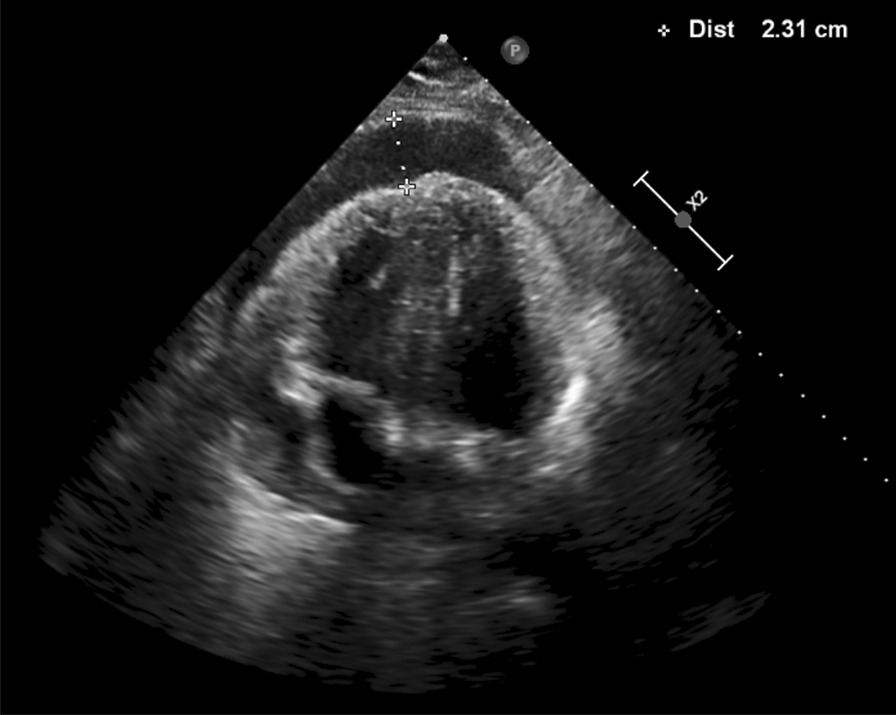
Transthoracic echocardiogram showing pericardial effusion (calipers)

A 44-year-old previously healthy male Causasian patient had a sudden onset of progressive dyspnea and wheezing in April 2019. The patient had no significant past medical conditions or illnesses, denied risky occupational exposure (worked in an office in the city), and reported social drinking and no smoking; family history was also unremarkable. Initial CT showed moderate pericardial effusion, bilateral pleural effusion (Fig. 1), and signs of pulmonary edema (Fig. 2). No other suspicious findings were reported. Transthoracic echocardiogram (Fig. 3) revealed signs of restrictive ventricular filling, with preserved ejection fraction (65%). In total, 825 mL of pericardial fluid was drained (negative for malignancy), and intravenous corticoid was started. In October 2019, after recurrent pericardial effusion, cytology was positive for neoplastic cells.

**Fig. 4:**
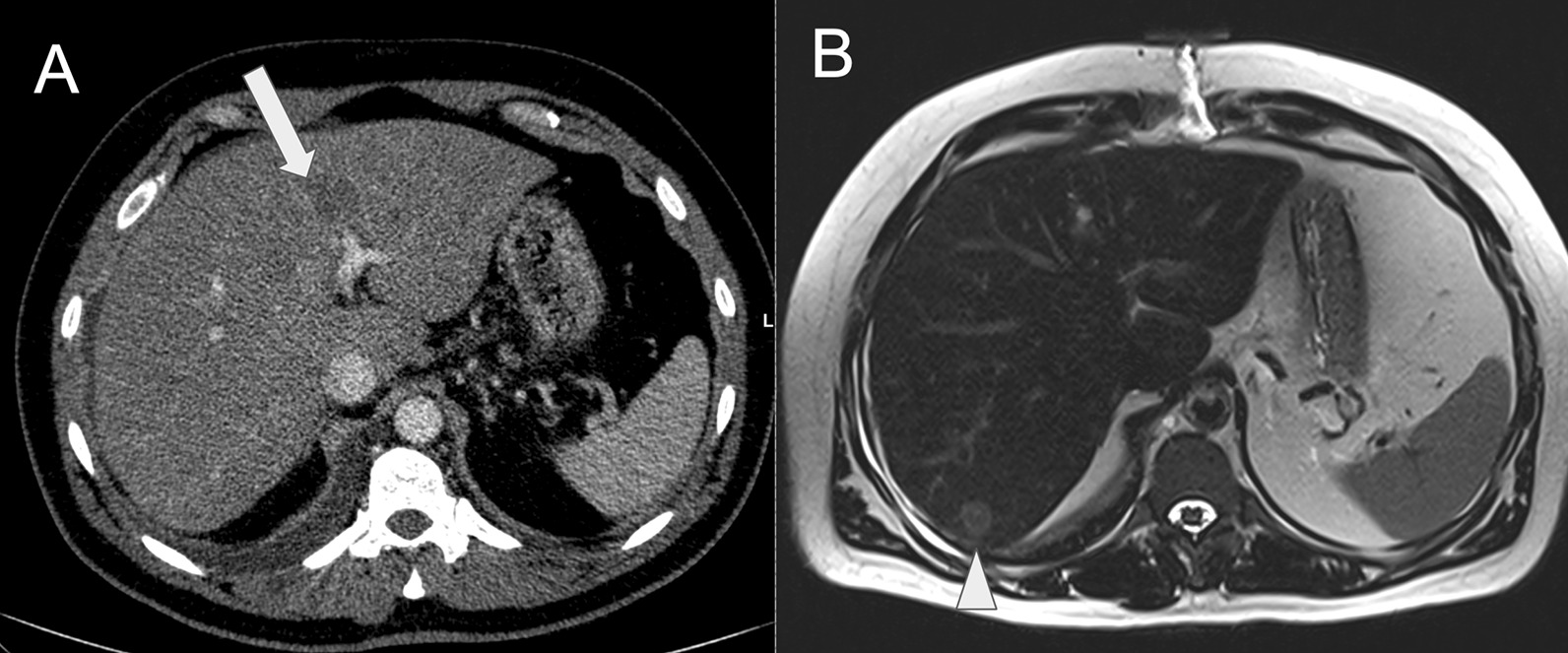
**A** Contrast-enhanced abdominal computed tomography showing an enlarged and heterogeneous liver, with ill-defined hypoattenuating indeterminate lesions (arrow). **B** Abdominal magnetic resonance (T2-weighted image) depicting a suspicious intermediate signal solid nodule (arrowhead) on right hepatic lobe

Repeated CT in October 2019 showed improvement in pulmonary congestion, signs of pericardiocentesis, and the appearance of indeterminate liver lesions, subsequently confirmed on magnetic resonance as solid nodules (Fig. 4). No conspicuous lesions were reported in intestines (although colons were not adequately distended). CT-guided liver biopsy was then performed, revealing adenocarcinoma of probable gastrointestinal origin (immunohistochemical analysis positive for CDX2, CK19, and CK20 and negative for CK7 and TTF1). Colonoscopy was carried out in November 2019, showing a vegetating and stenosing lesion of the sigmoid colon, biopsy-proven adenocarcinoma. The patient underwent laparoscopic rectosigmoidectomy and pelvic and retroperitoneal lymphadenectomy. Anatomopathological result was moderately differentiated invasive tubulopapillary adenocarcinoma. Peritoneal carcinomatosis was also observed. Pathological staging was pT2N2bM1.

Baseline tumor markers were CEA 363.8 ng/mL, CA 19-9 .5 IU/mL, and CA 125 41.3 IU/mL. First-line intravenous palliative chemotherapy was started with FOLFOX + bevacizumab, with clinical improvement and decreasing tumor markers. Genetic panel was obtained, indicating nonmutated KRAS and NRAS and microsatellite stability. Treatment response was assessed by CT scans in October 2020 and January 2021; disease was considered stable according to RECIST criteria. Currently, the patient receives chemotherapy with dose reduction, maintaining grade 1 thrombocytopenia without other laboratory changes and remaining asymptomatic (ECOG 0).

## Discussion and conclusions

We reported a rare case of a patient who presented with symptoms of cardiac tamponade and subsequent pulmonary edema due to malignant pericardial carcinomatosis and effusion, a unique first manifestation of an infradiaphragmatic (colon) cancer. Although other sites of metastatic disease were found, the pericardial involvement was the one that led to the initial clinical complaint and search for medical care, motivating the thoughtful investigation that brought light to the correct diagnosis.

Secondary pericardial involvement of gastrointestinal cancers is rare, and reports in the literature are scarce. In our review, we found only few cases of colon adenocarcinoma with secondary involvement of the cardiac serosa [4–6].

The diagnosis of *in vivo* cardiac metastasis may be difficult because most patients are asymptomatic. However, in 10% of cases, involvement of the pericardium can manifest as pericarditis, pericardial effusion, cardiac tamponade, or pericardial constriction (constrictive pericarditis), leading to an earlier suspicion of the diagnosis, especially in those with known neoplasia [8, 9].

Although cardiac manifestations are more common in patients known to have underlying malignancy, in some cases, including the one reported here, pericardial disease may be the first manifestation of malignancy [1, 10]. Thus, we must investigate occult neoplasia in individuals with persistent pericarditis who do not respond to antiinflammatory therapy and those with massive pericardial effusion (recurrent or hemorrhagic) or extensive cardiac tamponade [11, 12].

In patients with known neoplasia, it is worth emphasizing the importance of differentiating between progression of the oncological disease, metastasis to the pericardium, and complications secondary to an infectious process or to the antineoplastic treatment itself, since there are reports of acute pericarditis and pericardial effusion associated with the use of chemotherapeutic agents [1, 13]. Patients should undergo electrocardiogram, chest radiography, and echocardiogram as initial investigation. Additional imaging tests, such as CT scans or magnetic resonance imaging, should be performed to elucidate hidden neoplasia or restaging of a previously known cancer disease [8].

Definitive diagnosis requires pericardiocentesis with analysis of the cytological fluid and, in some cases, additional immunohistochemical study to assist in distinguishing between malignant cells and reactive mesothelial cells and to provide information about the probable tumor origin [9]. Although cytological fluid analysis is considered the gold standard of diagnosis and has lower false-negative rates than pericardial biopsy, negative cytology does not exclude malignancy in patients with a high suspicion of secondary pericardial involvement. In these cases, the investigation should be continued with pericardial biopsy and, preferably, pericardioscopy [9].

Once the diagnosis of neoplastic pericardial disease is established, and after specific therapy focusing on the underlying cardiac complications has been administered, an individual treatment plan should be developed that considers the volume of the disease, patient’s clinical performance, previous comorbidities, life expectancy, and existing antineoplastic therapies. Regardless of the therapy instituted, follow-up with the palliative care team is recommended because, even with treatment of the underlying neoplasm, the prognosis is poor [14]].

In conclusion, we believe this case report illustrates the importance of considering underlying neoplasia as a differential diagnosis in a patient with recurrent pericardial effusion, since it is a rare presentation of solid cancers from any site.

## Data Availability

All data generated or analyzed during this study are included in this published article.

## Abbreviations

CT: Computed tomography
CDX2: Cluster of differentiation X2
CK-19: Cytokeratin 19
CK-20: Cytokeratin 20
CK-7: Cytokeratin 7
TTF1: Thyroid transcription factor-1
CEA: Carcinoembryonic antigen
CA 19-9: Carbohydrate antigen 19-9
CA 125: Cancer antigen 125
FOLFOX: Chemotherapy scheme comprising oxaliplatin, 5-fluorouracil, and folinic acid
KRAS: Kirsten rat sarcoma viral oncogene
NRAS: Neuroblastoma rat sarcoma viral oncogene
RECIST: Response evaluation criteria in solid tumors
ECOG: Eastern Cooperative Oncology Group
